# Detection and Classification of Melanoma Skin Cancer Using Image Processing Technique

**DOI:** 10.3390/diagnostics13213313

**Published:** 2023-10-26

**Authors:** Chandran Kaushik Viknesh, Palanisamy Nirmal Kumar, Ramasamy Seetharaman, Devasahayam Anitha

**Affiliations:** 1 Department of Electronics and Communication Engineering, College of Engineering Guindy Campus, Anna University, Chennai 600025, India; drpnirmalkumar@gmail.com (P.N.K.); rseetharamanece@annauniv.edu (R.S.); 2 Department of Science and Humanities, Karpagam Institute of Technology, Coimbatore 641105, India; devasahayamanitha@gmail.com

**Keywords:** convolutional neural network, Django, melanoma, skin cancer, support vector machine

## Abstract

Human skin cancer is the most common and potentially life-threatening form of cancer. Melanoma skin cancer, in particular, exhibits a high mortality rate. Early detection is crucial for effective treatment. Traditionally, melanoma is detected through painful and time-consuming biopsies. This research introduces a computer-aided detection technique for early melanoma diagnosis-sis. In this study, we propose two methods for detecting skin cancer and focus specifically on melanoma cancerous cells using image data. The first method employs convolutional neural networks, including AlexNet, LeNet, and VGG-16 models, and we integrate the model with the highest accuracy into web and mobile applications. We also investigate the relationship between model depth and performance with varying dataset sizes. The second method uses support vector machines with a default RBF kernel, using feature parameters to categorize images as benign, malignant, or normal after image processing. The SVM classifier achieved an 86.6% classification accuracy, while the CNN maintained a 91% accuracy rate after 100 compute epochs. The CNN model is deployed as a web and mobile application with the assistance of Django and Android Studio.

## 1. Introduction

Skin plays an important role in regulating body temperature and in protecting us from the environment, including sunlight [1]. However, many skin diseases, including malignant tumors caused by genetic factors, can occur. Cutaneous malignancies such as squamous cell carcinoma, melanoma, and basal cell carcinoma pose a threat with low survival rates for affected individuals [2,3]. These three types of cancer together account for more than 3.5 million diagnoses each year, including melanoma [4]. Melanoma, one of the most dangerous types of cancer, occurs when melanocytes in the layers of the skin, hair, eyes, and other organs become immune, resulting in a malignant tumor [5]. Melanoma growths usually appear black or brown but may also appear red, pink, or purple [6]. Research conducted at a British university has shown that 86% of melanomas are caused by exposure to ultraviolet (UV) radiation. The risk of developing melanoma doubles when a person has had up to five sunburns [4]. Melanoma can lead to skin cancer, especially in people with fair skin. However, early detection of melanoma before symptoms appear can lead to a 90% cure rate [7]. Skin cancer is usually diagnosed by biopsy, but the process is slow and not always accurate. Noninvasive techniques such as macroscopic and dermoscopic imaging have become indispensable for dermatologists in skin evaluation. Dermoscopic photography provides high-resolution images of the skin that are invisible to the naked eye, and it reveals deeper skin layers, thus improving skin detection [8]. Dermoscopic images help in the diagnosis of skin cancer. Over the past few years, computer vision and digital image processing have become important tools to speed up visualization and reduce human errors [1]. Studies have explored the use of imaging techniques to identify non-melanoma tumors [9] and the use of deep learning to identify melanoma markers from dermoscopic images [10]. Dermoscopic imaging provides a method to diagnose skin cancer. In recent years, computer vision algorithms and digital image processing have played an important role in accelerating vision and reducing human error. Technology and advances such as image processing and artificial intelligence can help dermatologists detect the presence of melanoma, thus improving melanoma detection and reducing suspicion related to biopsies [11]. Another computer-aided diagnosis (CAD) method has been proposed for cancer diagnosis [12,13]. Traditional computer vision techniques are used to analyze various characteristics such as size, color, and texture to identify cancer cells. However, it can be difficult to distinguish images based on these features alone. Today, artificial intelligence (AI) is solving these problems. The research on skin cancer detection is ongoing [14] and deep learning algorithms have been used to identify melanoma markers from dermoscopic images [15]. The abstraction capacity of neural network layers allows convolutional neural networks (CNNs) to automatically extract high-level features (such as semantics and patterns) and low-level behaviors (such as edges and shapes). This ability makes this method more accurate in diagnosing skin melanoma [16]. Considering the severity of non-melanoma tumors, various cancer diagnostic methods have been developed that make CNN-based diagnosis essential for today’s treatment [17]. CNNs outperformed all experts on both diagnoses, showing that awareness can help skin cancer groups as well as dermatologists [18]. Thanks to artificial intelligence and deep learning, the device has the potential to replace dermatologists and allow smartphone users to access rare medical services for diagnosis [19]. The diagnosis of skin cancer involves four main areas: dermoscopic image data acquisition, image pre-processing, segmentation, and feature extraction. Multiple classification methods are used, including artificial intelligence and deep learning-based algorithms, to improve classification results. In general, SVM classifiers have been found to be more accurate and less computationally expensive, taking approximately 5 min to gather an insight [20]. The data are collected from dermoscopic image data acquisition, image pre-processing, segmentation and feature extraction, which are the four elements of the diagnosis of skin cancer. Many methods have been combined to improve the classification results, including the use of artificial intelligence and deep learning algorithms. Taking all the points into account, the SVM classifier offers high accuracy and low performance, taking about 5 min to complete the evaluation [21]. The techniques for diagnosing various melanomas by learning can be divided into segmentation and classification. The next section will discuss the use of techniques such as decision skinning (and the various types of decision trees and support vector machines), logistic regression, stochastic gradient descent, random forests, and naive Bayes. This technique can be used to effectively identify skin diseases. The results show that among various classification methods, LR, NB, and SVM have the highest accuracies at 92%, 92%, and 89.6%, respectively [22]. According to the ABCD principle, seven different factors, including structure, size and diversity, have been proposed to identify melanoma at an early stage. The analysis of 200 images was included in the SVM and ANN classification algorithms, and the accuracy reached 94% and 92%, respectively. Dermatologists may benefit from incorporating this approach into their clinical decision-making processes. However, as the amount of information increases and classification improves, neural networks will be used for melanoma classification [23]. A compact and inexpensive skin cancer screening device has been developed to help detect melanoma in a timely manner. The main function is an emergency alert designed to help users avoid sunburn. Often an additional algorithm must be included to calculate the time required for epidermal heating. Experimental results show that the proposed design is effective and provides the best classification [24]. Two artificial intelligence methods, namely the support vector machine (SVM) and convolutional neural network (CNN), were used to detect skin cancer, and the experimental results are presented during the modeling. These processes include image acquisition, image preprocessing, segmentation, feature extraction, training and testing, and classification, all of which play an important role in determining the location of the wound on the skin.

## 2. Materials and Methods

**Figure 1:**
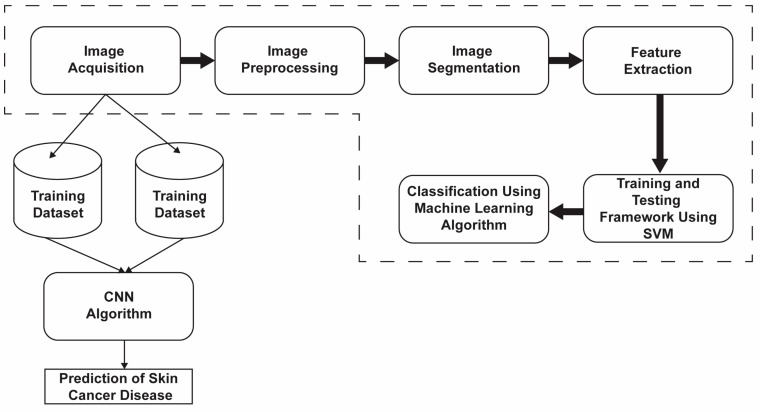
Block diagram of the proposal.

The following part portrays the dataset assortment and preprocessing, followed by an examination utilizing SVM and CNN techniques. The information is divided into preparing and testing assortments and can be perused by means of a Jupyter-fabricated notepad utilizing the operating system library capability [25]. Figure 1 shows the block diagram of the proposed item.

Imaging Recognition Dataset: The ISIC (International Skin Imaging Collaboration Laboratory) database contains images of benign, malignant, and normal skin lesions. The data are available in the ISIC repository, and Kaggle also hosts different images of different sizes. A total of 673 images were collected in the training and testing.

Data Preprocessing: Skin disease images are preprocessed to extract important information from noisy images, remove noise, and create high-quality images [26]. In order to obtain a good image, the skin image must first be processed to extract important information from the complex and noisy image. Preprocessing removes unwanted objects, such as artifacts, hair, skin pigmentation, and air bubbles, using the following steps:

1. Resize the fill in the image.
2. Convert RGB images to grayscale images. Grayscale images enable the automatic processing that is often used in digital systems.
3. Put the grayscale image through five filtering processes to reduce noise, determine the noise reduction target, detect and remove hair, and heal skin damage.

These include non-local averaging and block-matching 3D filters. The results were analyzed using the image size parameter. The preprocessing performance indicators were: the MSE value (mean square error), PSNR value (peak signal-to-noise ratio), SSIM value (structural similarity index), and other performance indicators; the universal quality index (UQI) was used to evaluate the original image and to reconstruct the image that was created. Various prefiltering techniques were compared with the performance metrics above to determine the best method of removing database noise.

Mean Squared Error (*MSE*): Equation (1) calculates the *MSE* as the sum of the squared error between the compressed image and the original image. This is a general guide, and the closer it is to zero the better.

(1) $$MSE=\sum \sum \left(\frac{error*error}{M*N}\right)$$

Among them, the image was not correct before re-rendering the image after the game. *M* is the number of lines in the image file, and *N* is the number of sections in the image file.

Peak Signal-to-Noise Ratio (*PSNR*): The *PSNR* measurement measures the ratio of the maximum signal to the noise in the input image and the output filter produced by the input processing. The higher the *PSNR* value, the better the filtering. This *PSNR* value is calculated according to Equation (2) and is a measure of the difference between two video images.

(2) $$\;\;\;\;\;\;PSNR=20.\log_{10}\frac{MAX_{I}}{\sqrt{MSE}}$$

where *MAX_I_* indicates the greatest worth of the pixel.

Structural Similarity Index (*SSIM*): The structural similarity index (*SSIM*) calculates the amount of structural similarity between two images. At the same time, the difference in brightness and contrast ratio can be calculated using Equation (3). It also includes many other important aspects of perception, such as the fact that the range of the *SSIM* index is −1 to 1. For the same image, the value is always 1.

(3) $$SSIM\left(x.y\right)=\frac{\left(2{µ}_{x}{µ}_{y}+C_{1}\right)\left(2\sigma_{xy}C_{2}\right)}{\left({µ}_{x}^{2}+{µ}_{y}^{2}+C_{1}\right)\left(\sigma_{x}^{2}+\sigma_{y}^{2}+C_{3}\right)}$$

Universal Quality Index: The index is created by modeling each image’s degradation based on a combination of three factors: detail loss, brightness degradation, and texture degradation.

Segmentation: The preprocessed images are segmented to identify specific areas of the skin. The data in this study were segmented using a combination of watershed segmentation and maximum entropy thresholding. Maximum entropy: entropy is the measure of variation or variety in an image. The maximum entropy is used to determine the binarization threshold. The threshold obtained from the raw image is used to encode the maximum entropy and to distinguish between the foreground and the background. This process produces a binary image in which skin lesions stand out against the background. Entropy calculations are also used for initialization. The maximum entropy threshold maximizes interclass entropy by increasing the information separation between the objects and the background, as described in Equation (4).

(4) $$H=\sum_{pk}log_{2}\;\left[p\left(k\right)\right]k$$

where *k* is the number of darkness areas, and *p*(*k*) is the probability associated with darkness *k*.

Morphological operations: Morphological operations are used to improve image quality by smoothing edges, removing small pixels, removing light artifacts in images, and filling empty holes. This function is used to create digital images and improve the quality of dermoscopic images. The registration function is used to fill in gaps and smooth edges. Morphological dilatation and erosion processes are performed to create the final image.

Feature Extraction: Extracting features from segmented skin lesions reduces the amount of initial data by measuring specific features that distinguish one type of input from another. Feature extraction provides important skin information, including math, images, data, and evidence. This process converts the amount of data into a record that is easy to analyze with high performance. In our study, we extracted specific and unique features that distinguish malignant melanoma from benign melanoma. The data collected are used to identify skin lesions.

First-order statistical features: First-order labels are widely used and are simple indicators that describe the distribution of pixels used in the image area defined by the cover.

Grayscale (minimum and maximum weight): Conditions associated with the central and most important weak level in the covered area (Equation (5)).

(5) $$f_{min}=\min\left\{f\left(x,y\right)\right\}\;;\;\;\;f_{max}=\max\left\{f\left(x,y\right)\right\}$$

Average: Equation (6) computes the typical dim level force inside the region of interest.

(6) $$\mu =f_{1}=\underset{i}{\sum }iH_{i}$$

Standard Deviation = Variance ½: Standard deviation measures the amount of deviation or spread from the mean and is given by Equation (7).

(7) $$\sigma =f_{2}=\sqrt{\sum_{\iota }{\left(i-\mu \right)}^{2}H_{i}}$$

Coefficient of variation: This is the proportion of the standard deviation to the mean, as determined by Equation (8).

(8) $$f_{cov}=\frac{\sigma }{\mu }$$

Histogram Width: The width of the histogram addresses the stretch determined by Equation (9).

(9) $$f_{hw}=f_{90}-f_{10}$$

Gray level size zone matrix: Grayscale zones in a picture. The quantity of associated pixels with the equivalent grayscale force is known as a grayscale zone. As opposed to GLCM and GLRLM, GLSZM is pivot free and just a single grid is figured for all headings inside the region of interest. In GLSZM *p*(*i*,*j*), the (*i*,*j*)th component compares the quantity of zones in the picture with the dim level and size.

- *Ng* shows the quantity of the various power values.
- *Ns* shows the quantity of the individual zones.
- *P*(*i*,*j*) is the size zone framework.
- $Nz=\overset{Ng-1}{\underset{i=0}{\sum }}\overset{Ns-1}{\underset{j=0}{\sum }}P\left(i,j\right)$ is the number of zones in the ROI.

Grayscale Variance (*GLV*): This measures the adjustment of the dim level in the picture and is given by Equation (10).

(10) $$f_{GLV}=\sum_{i=0}^{N_{g}-1}\sum_{j=0}^{N_{s}-1}p\left(i,j\right){\left(i-\mu \right)}^{2}$$

Zone Size Entropy (*ZSE*): This estimates the vulnerability/irregularity in the dissemination of zone sizes and dim levels and is given by Equation (11).

(11) $$f_{ZSE}=-\sum_{i=0}^{N_{g}-1}\sum_{j=0}^{N_{s}-1}p\left(i,j\right)\log_{2}\left(p\left(i,j\right)+\varepsilon \right)$$

Gray Level Co-occurrence Matrix: The GLCM is a robust technique for extracting image features by mapping the gray level co-occurrence probabilities based on the spatial relationships of the pixels in various angular directions [26,27,28,29]. GLCM is essential in texture analysis, where fine details from an object are captured. It involves two pixels: a neighboring pixel and a reference pixel. The statistical approach of texture analysis takes into account the spatial arrangement of the pixels. The GLCM of the entire lesion describes the texture by assessing how frequently pairs of pixels with specific brightness values and orientation appear in an image [30,31].

- *P*(*i*,*j*) is the co-event framework.
- *N* is the number of dark levels.
- *p_x_*_+*y*_(*k*) = Σ Σ*P*(*i*,*j*); *p_x_*_−*y*_ (*k*) = Σ Σ*P*(*i*,*j*); µ*_x_*_+*y*_= Σ*k_px_*_−*y*_ (*k*).
- *p*(*i*,*j*) is the normalized co-occurrence matrix and is equal to $\frac{p\left(i,j\right)}{\sum_{i=0}^{N-1}\sum_{j=0}^{N-1}P\left(i,j\right)}$.

Inverse Difference Moment: This is represented by Equation (12); it quantifies the local homogeneity of an image.

(12) $$f_{IDV}=\sum_{i=0}^{N-1}\sum_{i=0}^{N-1}\frac{p\left(i,j\right)}{1+{\left(i-j\right)}^{2}}$$

Sum Average: The sum average is calculated using Equation (13); it evaluates the relationship between occurrences of pairings with lower and higher intensity levels.

(13) $$f_{SV}=\sum_{k=1}^{2N-1}kp_{x+y}\left(k\right)$$

Contrast Entropy: Contrast entropy serves as a metric for quantifying the unpredictability in the local intensity value differences; it is calculated using Equation (14).

(14) $$f_{DE}=-\sum_{k=0}^{N-1}p_{x-y}\left(ik\right)log\left[p_{x-y}\left(k\right)\right]$$

Mathematical Features: Mathematical properties include points, lines, curves and surfaces, which may include edge features, shape features, or geometric features. Mathematical properties are used to calculate the area and perimeter, showing the size of the lesion in pixels and the perimeter in pixels.

Boxplot: A boxplot is a graphical representation that helps in the visualization of the distribution of the values in the data. It is especially useful for comparing groups or sets of data.

Classification: Various criteria are used to classify skin diseases as malignant or benign. Machine learning is often used for this purpose. Common tools include support vector machine (SVM), nearest neighbor (KNN), naive Bayes, and neural networks. In this study, an SVM classifier was used, and features were extracted directly into the classifier.

Support Vector Machine (SVM): SVM is a supervised machine learning algorithm used for the classification of tasks with many groups. It outperforms other machine learning methods in solving multi-class problems. SVM effectively determines the boundary by optimizing the boundaries in the dataset. Hyperplanes are used to define the boundaries to divide the data into different groups. In this case, SVM classifies skin cancer images as benign or malignant. SVM uses a cost-sensitive classification system and enables integration to build multiple machine learning models by selecting parameters. The model is scaled and trained using a simple SVM classifier. Radial basis function (RBF) kernels are commonly used on nonlinear datasets. Diagnosing melanoma is a type II problem with a possible output value of “0” for benign disease and a possible output value of “1” (positive) for malignant disease. Therefore, a linear SVM model was developed. It examined approximately 50 images of benign and malignant diseases. The course was evaluated using 70% of the training material and 30% of the testing material.

### Model Evaluation

**Figure 2:**
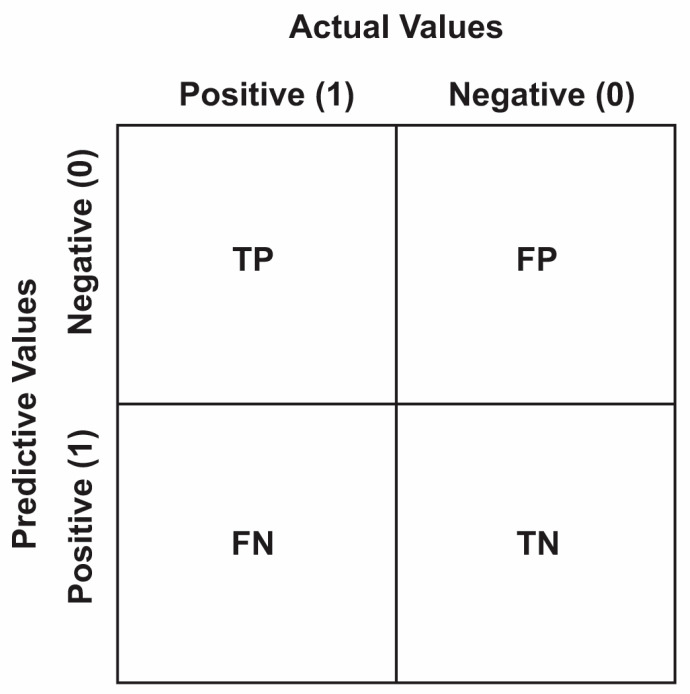
Confusion Matrix.

The confusion matrix (Figure 2) is a table often used to represent the performance of the classification model on known test data. From here, various metrics, such as accuracy, recall, precision, and F1 score, can be calculated to evaluate the effectiveness of the classification model.

True Positive (*TP*): A positive sample is correctly predicted.

True Negative (*TN*): Negative class instances are predicted as negative instances.

False Positive (*FP*): A negative class is incorrectly predicted as positive (also known as a “Type I error”).

False Negative (*FN*): A positive sample is incorrectly predicted as negative (also known as a “Type II error”).

*Accuracy*: This refers to all the records classified by the separator. Classifier accuracy is defined as the percentage of test groups classified by the model [31], as described in Equation (15).

(15) $$Accuracy=\frac{TP+TN}{TP+TN+FP+FN}\times 100\%$$

*Sensitivity*: The response is characterized by a small number of useful perceptually good groups, as determined by Equation (16).

(16) $$Sensitivity=\frac{TP}{TP+FN}\times 100\%$$

*Specificity*: This shows how often the test or symptoms lead to correct (including negative) decisions in healthy patients [32]. Equation (17) is used to specify the properties.

(17) $$Specificity=\frac{TN}{TN+FP}\times 100\%$$

*Precision*: The big event scene is back. The condition is determined using Equation (18).

(18) $$Precision=\frac{TP}{TP+FP}\times 100\%$$

*F-measure* is defined as the relationship between precision and sensitivity, which improves the use of events (Equation (19)).

(19) $$F-measure=2*\frac{precision*sensitivity}{precision+sensitivity}\times 100\%$$

Model Interpretability using Grad-CAM: In this section, we take a closer look at the interpretation of predictive models using Grad-CAM techniques. Model interpretation is essential to understanding and to confidence in the application skills, and Grad-CAM provides a powerful tool to identify the input fields that contribute the most to this classification decision model. Grad-CAM is a technique that identifies key regions in an input image by calculating the gradient of the target group relative to the final convolutional layer map. This allows us to see which of the individual displayed images has the greatest impact on the decision model. We use Grad-CAM when selecting representative test samples to gain more insight into our model’s classification process. This involves calculating the gradient of the target score based on the specific map of the last convolution layer in our model. Combining Grad-CAM visualization and results analysis explains how the model focuses on specific aspects of the input data when making predictions. For example, if the model segments the images, display the Grad-CAM heat maps on some of the sample images to show which areas of the image the model pays the most attention to when segmenting. The implications of this finding are then discussed. Grad-CAM indicates whether regions follow human assumptions about the distribution of values. The general results of the interpretation obtained with Grad-CAM are discussed in the context of the application. Understanding this is important in real situations. We conclude this section by showing how Grad-CAM integration improves the interpretation model and why it is important for publication. Demonstrating the model’s ability to provide transparent and understandable results is important for the field of artificial intelligence and ensures that the model’s predictions are reliable and respond robustly to its limitations. By including this section on model interpretation using Grad-CAM, the paper not only provides a powerful classification model but also contributes to the growing body of research conducted to make AI systems transparent and accountable. This development increases the functionality and usability of the report.

Convolutional Neural Network (CNN): Unlike artificial intelligence, the CNN extracts visual features from raw pixel data. An n x n grid of points/channels (where n is not the meaning of the resulting image) is convolved with the image data to form a first-level convolutional inclusion frame of the image dataset. Semi-transparent network data from the device are used as the main communication channel. This stage works independently of the R, G, and B layers of the image and creates the main meaning by combining the expressions recognized as (i,j). As the number of channels used for data increases, CNNs can classify more content, but the preparation time also increases. Therefore, the best number of blocks to extract important points in terms of image features is determined. This is all performed in Python using TensorFlow and Keras libraries. TensorFlow is an open source library and one of the best deep learning architectures. Keras is deep learning. Keras runs on top of Tensor-Flow; so, it is not a candidate for TensorFlow. Keras uses a very simple sentence model to create a brain organization, transforms text into a ten-gram flow model, and combines this with the power of text to power the entire learning tool [32,33,34].

Proposed Approach: The convolutional neural network (CNN) is a type of neural network widely used for object detection, image segmentation, and semantic segmentation. Convolutional layers extract complex nonlinear features from images. Proposed method: the convolutional neural network (CNN) is a neural network widely used for object detection, image segmentation, and semantic segmentation. Convolutional layers extract complex nonlinear features from images. The pooling layer is further divided into a maximum pooling layer and an average pooling layer to reduce the number of parameters and speed up the CNN training process. The fully connected layer collects the features learned from the previous convolution process and then presents the final result of the output process. The TensorFlow function library is used to initialize multiple CNN models throughout the training process. This involves loading training data into the model, fine-tuning it, and evaluating how the changes affect the model’s performance. A table showing specific parameters and their values for various models, using the same data during analysis is created. Big data is searched for analysis using pretrained CNNs designed for skin cancer diagnosis. A pretrained CNN was used to identify skin cancer, and pretrained AlexNet and VGG-16 architectures for skin recognition were used to distinguish between dermoscopic images [35,36]. In this study, we evaluate ISIC data using VGG-16, AlexNet, LeNet, and customized CNN architectures. The model is imported from the Keras framework into TensorFlow and trained using the same data using the same support method. VGG-16 is a convolutional neural network, it has 13 convolutional layers, 5 pooling layers, 3 full layers, and softmax layers. Due to its hardness, this model is famous for handling deep water. However, due to its large scale, a lot of training is required. AlexNet is the first integrated network to support GPUs to increase performance. Its architecture includes 5 convolutional layers, 3 max-pooling layers, 2 normalization layers, 2 sum layers, and 1 softmax layer. Each convolution layer contains a convolution filter and a ReLU nonlinear activation function, and the pooling layer is used for maximum pooling. LeNet was one of the first neural networks developed for deep learning. The LeNet-5 CNN architecture consists of 7 layers, including 3 convolutional layers, 2 subsampling layers, and 2 fully connected layers. The CNN design model consists of 11 layers, including a recursive layer, convolutional layer, layer, and thickness layer. The model is built layer by layer using a series of operations in TensorFlow. The aim is for the model to use the prepared image and to try to use the suggested image. First, the rescale layer rescales the top portion of the RGB channels in the infographic [from 0.255 to 0.1].

**Figure 3:**
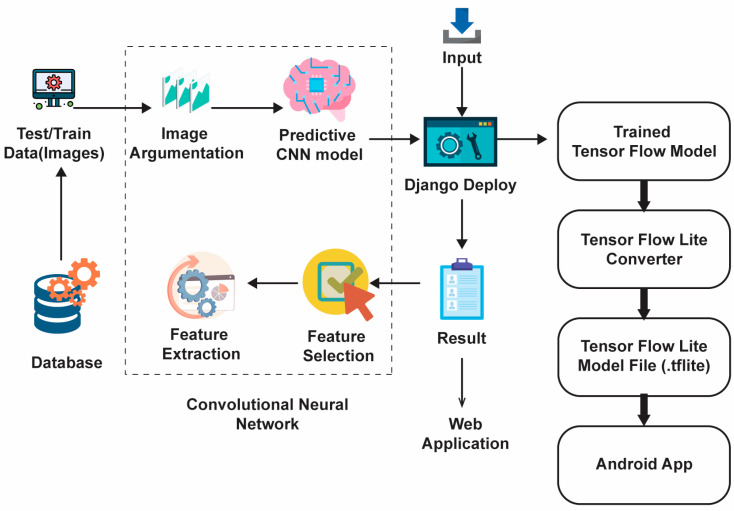
Workflow for web and Android development.

- The enhanced data are then used to create new images with rotation, cropping, scaling, transforming, and horizontal and vertical features. These mathematical changes were made without altering the original data, and they increased the accuracy of the model used. The rotation changes the image by rotating it clockwise or counterclockwise at random angles. Flipping is the process of turning an image along its horizontal or vertical axis. Translation moves the image along the x or y axis. The clipping replaces the image content with a random page. Resizing increases the size of the image. After all the transformations are performed, each image is normalized. When the dataset is increased, it is divided into training and testing sets. During training, 70% of the dataset is used for model learning, training, and validation, and 30% is used for model testing and evaluation. Image file creators help with this.
- Both the Conv2D layer and the max pooling layer are used to extract features and reduce the dimensions. The output channels of the two Conv2D layers are set to 32 and 64, respectively. ReLU is used in the activation function to mitigate the vanishing gradient problem and to expedite the learning process. The padding is set equally for all the layers to ensure that the output dimensions match the input dimensions.
- Following the pooling layer is a ReLU function that down-samples the convolutional feature map while preserving important feature information. A commonly used max-pooling technique employs an NxN pooling filter, sweeps across the data feature map, selects the highest value, and discards all other values to generate the output feature map.
- After the two Conv2D layers and pooling layers, a dropout layer with a dropout probability of 0.2 is added to constrain the composition and prevent overfitting. Finally, one even coat and two thick coats are applied. The dense layer contains 3 neurons corresponding to the patient’s skin.
- The project compared four models and chose the most reliable for web and mobile use. To make the comparison fair and efficient, each model was trained with a batch size of 32 and optimized using Adams optimization with categorical cross-entropy as a failover.
- The model was trained once more (100 times) to reduce the error rate. Each period includes training, validation, and training and validation. The prior learning is 0.0001, and the measurement accuracy is correct.
- The graph showing accuracy and loss is always seen. The success rate is approximately 91%. These criteria determine whether a skin lesion is benign, malignant, or normal over time. *keras.models.save_model()* is used to save the models and to deploy them to local servers and mobile applications using Django, a powerful web development framework. The overall working of the web and Android development is shown in Figure 3.

Django: Django is a Python web development framework for quickly building secure, maintainable websites. Developed by experts, Django removes much of the complexity from web development, allowing one to focus on building applications instead of wasting time. It is open-source and has a strong community, great value, and many free and paid options.

Use Android Studio to develop Android applications: It is recommended to use the Android Studio integrated development environment (IDE) for Android application development. Android Studio includes new features that make Android app development more efficient. It is based on IntelliJ IDEA, an integrated Java programming environment, and includes its own code editing and development tools.

Model transformation: During model transformation, the TensorFlow Lite model in the Android app receives data, analyzes the data, and makes predictions based on model information. There must be a special time for it to run, and the data given to the model must be in special files called tensors. When the model analyzes the data (called “inference”), it creates another prediction tensor that can be presented to the user or used for other purposes in Android applications. The TensorFlow Lite converter takes a TensorFlow model and transforms it into a TensorFlow Lite model, a non-destructive optimization identified by the “.tflite” file extension. One can load a saved model or change the design directly in the code. Designs can be modified using the Python API or command line tools.

## 3. Results and Discussion

**Figure 4:**
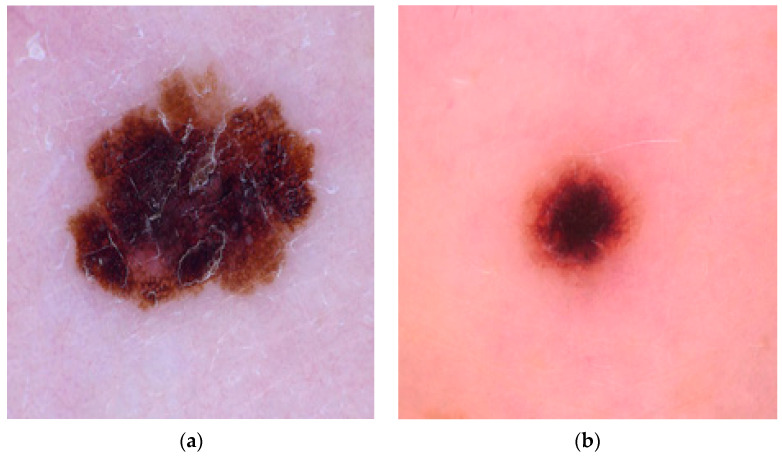
(**a**,**b**) Dermoscopic image: (**a**) malignant, (**b**) benign.

The dermoscopic images obtained from the ISIC dataset were initial processed, segmented using various image processing techniques, and classified using AI methods. The dermoscopic images collected from the dataset are shown in Figure 4a,b.

**Figure 5:**
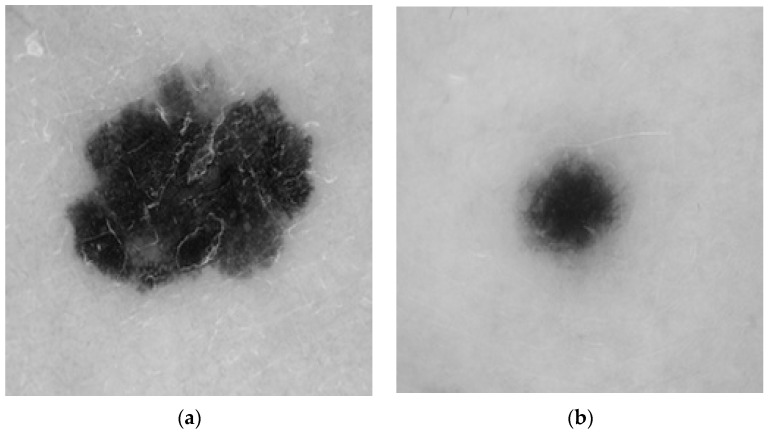
(**a**,**b**) Grayscale image: (**a**) malignant, (**b**) benign.

**Figure 6:**
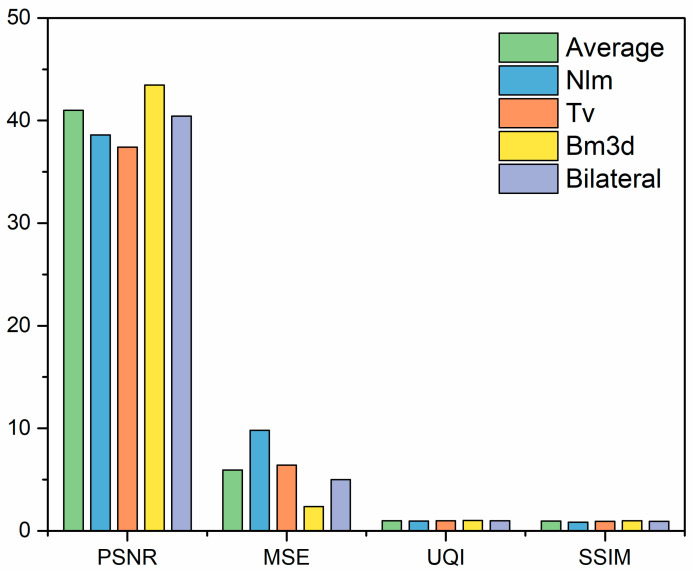
Graphical representation of five filters.

The image collection was initially converted to 256 × 256. Grayscale images are easier and faster to process than color images. This conversion converts the RGB image into a grayscale image. Figure 5a,b show grayscale images. The graphs of five filters show that block-matching 3D filtering effectively reduces noise and eliminates artifacts in the dermoscopic images compared to the other filters. Figure 6 gives representations of these five filters.

The mean square error between the original image and the reconstructed image is small, indicating that the image was restored correctly. In addition, the maximum signal-to-noise ratio (PSNR) value obtained is also quite good, which means that the image quality was restored. The system similarity index (SSIM) and universal image quality index (UQI) values close to 1 indicate that the image was restored. The performance of five prefilters for noise reduction and hair removal was evaluated and compared.

**Figure 7:**
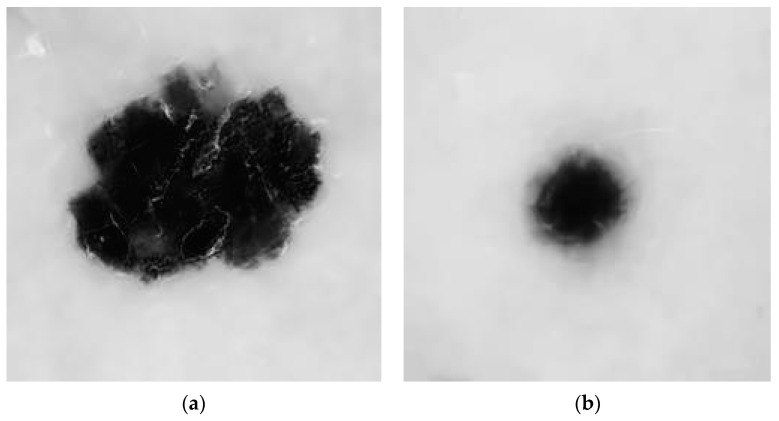
(**a**,**b**) Block-matching 3D filtered image.

**Figure 8:**
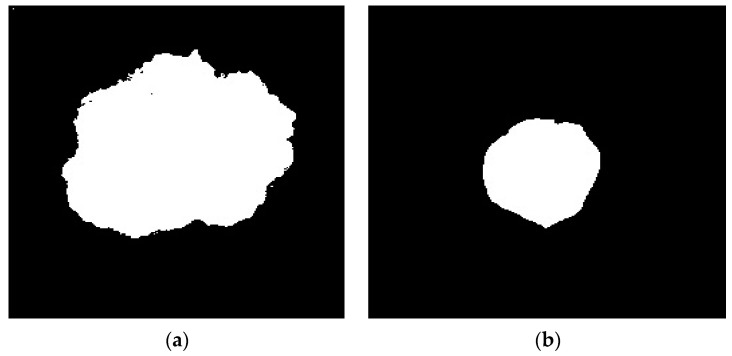
(**a**,**b**) Binary mass lesion.

Figure 7 shows the 3D image separation, which reduces noise and hair quality. The preprocessed images were segmented using the maximum entropy thresholding technique. The maximum entropy threshold is used to convert the reference image into a binary image. The obtained entropy value is used to evaluate the image, and an entropy calculation is used in this process. To create a binary mask, all the white pixels in the corners are replaced with black pixels. Table 1 provides information about the entropy and thresholds. Figure 8a,b show the binary masks that result in segmentation using the maximum entropy thresholding method. Figure 7 shows the corresponding 3D segmentation image, which reduces noise and hair quality. The preprocessed images were segmented using the maximum entropy thresholding technique. The maximum entropy threshold is used to convert the reference image into a binary image. The obtained entropy value is used to evaluate the image, and an entropy calculation is used in this process. To create a binary mask, all the white pixels in the corners are replaced with black pixels. Table 1 provides information about the entropy and thresholds. Figure 8a,b show the binary masks that result in segmentation using the maximum entropy thresholding method.

**Figure 9:**
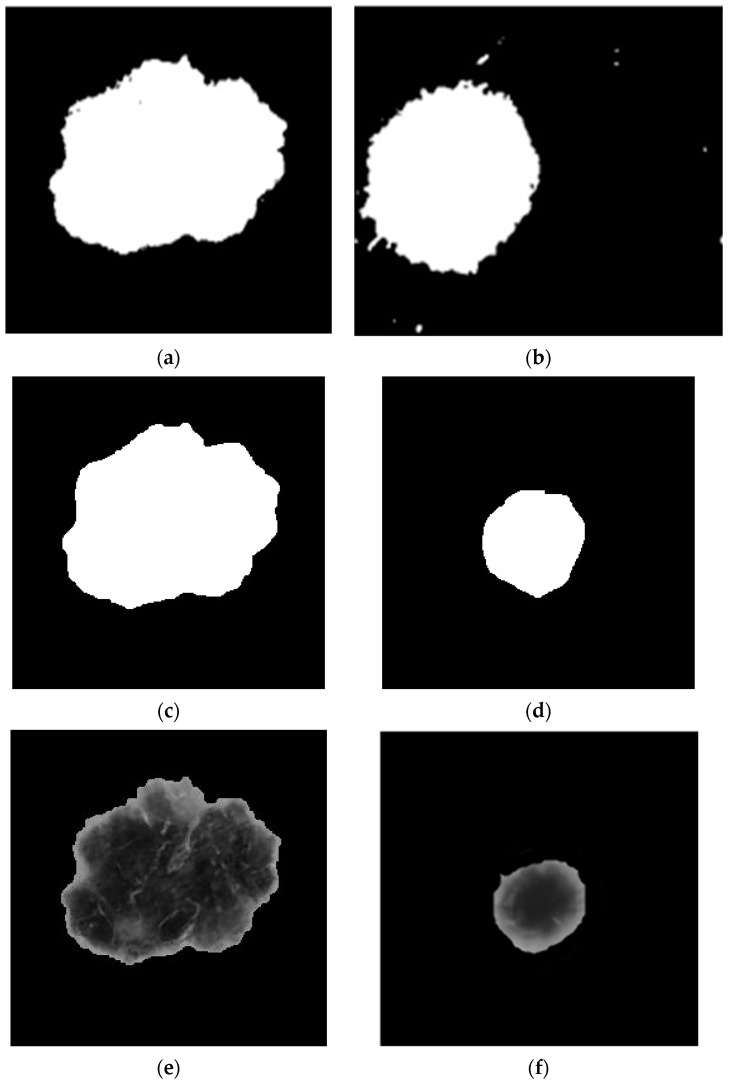
(**a**,**b**) Binary lesion mask formed by maximum entropy thresholding. (**c**,**d**) Morphological operation. (**e**,**f**) Final segmented image.

**Figure 10:**
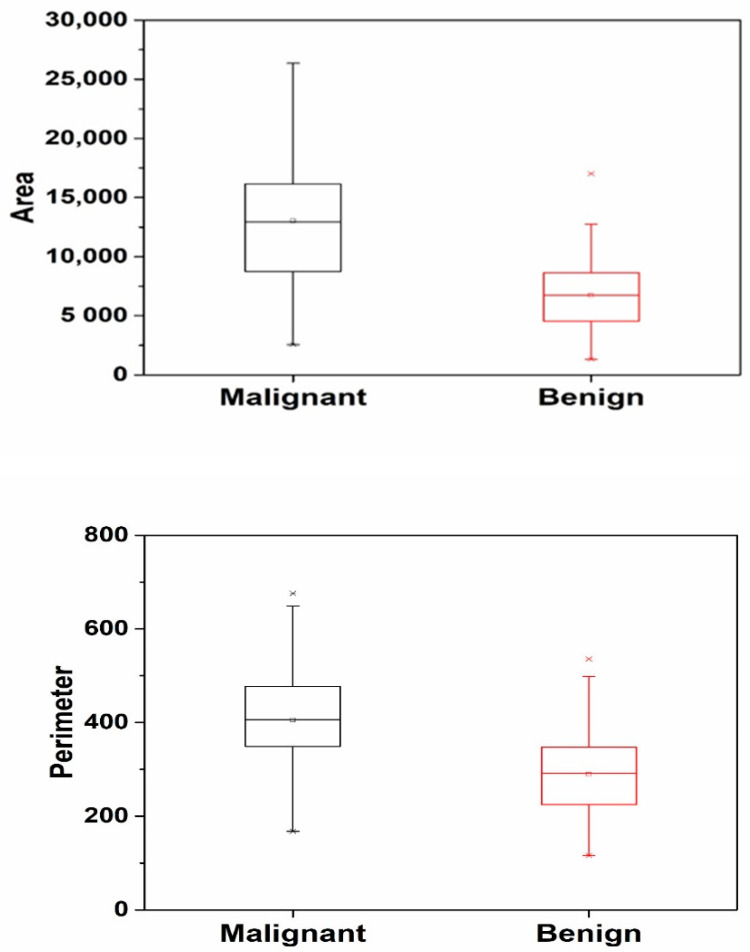
Boxplot.

Thresholding can cause jagged edges in the resulting image. Figure 9a,b show the binary lesion mask created using the maximum entropy threshold. Apply morphological filtering to smooth the edges, remove the noisy pixels that distort the light pattern associated with the image edges, and fill and unfold the image area to obtain a binary morphological image, as shown in Figure 9c,d. To create segmented lesions, a binary lesion mask is created and applied to the previous image. Figure 9e,f show the segmentation results. The mathematical properties, including GLCM, GLSZM, and the product statistics, were extracted from the segmented organisms and recorded in an Excel spreadsheet. The distribution of the data between the feature space and the surrounding malignant and benign datasets is presented as a boxplot showing the differences between the two datasets (see Table 2 and Figure 10). The mean and standard deviation of the features obtained from the malignant and benign images were also compared.

All the collections were sent to the SVM classifier, where 70% of the data were used for training and 30% for testing. Information on the distribution of the isolates is found in Table 3 and Table 4.

**Figure 11:**
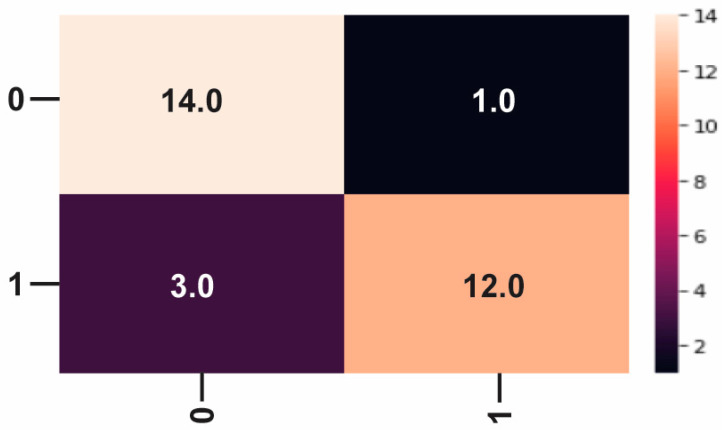
Confusion matrix of tested data.

Although some beautiful images were incorrectly classified as negative, the ability of the classifier to identify infected individuals is high; it has a 92% accuracy rate in distinguishing between bad problems. However, the process by which disease, especially malignancy, can be detected is not accurate. It was identified as benign in 82% of patients but did not classify three benign images, which is concerning. Overall, the SVM classifier achieved an accuracy of 86.6%. The confusion matrix of the test data is shown in Figure 11.

Table 5 shows the model assessment boundaries, like awareness, particularity, and exactness, for the SVM classifiers.

**Figure 12:**
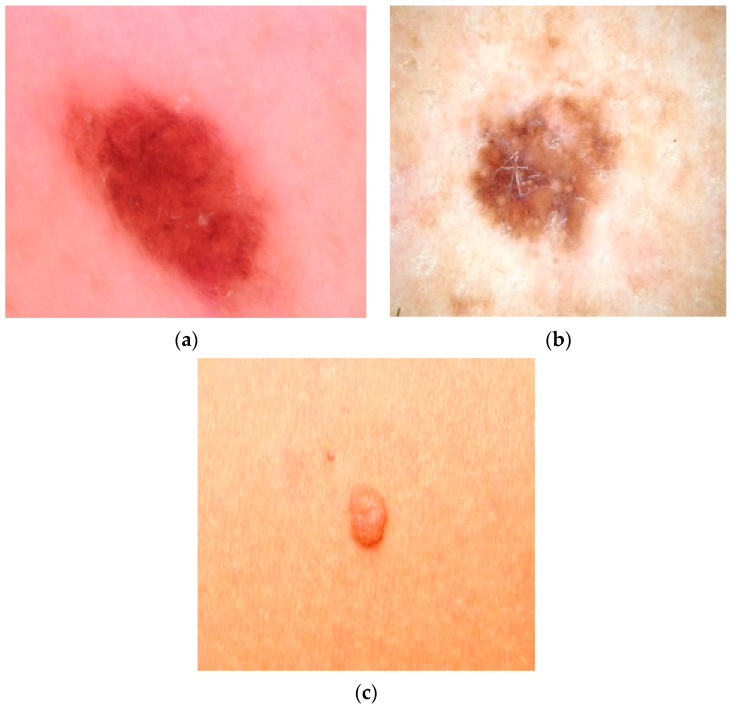
(**a**) Dermoscopic image of malignant growth. (**b**) Dermoscopic image of benign growth. (**c**) Dermoscopic image of normal growth.

The dermoscopic images were obtained from the open source ISIC dataset (International Skin Imaging Collaboration). The captured images are categorized based on the structure and texture related to the main features of these images. Experience using convolutional neural networks (CNN) and deep learning has led to great success in classifying different types of cancer. We applied a variety of neuron-level and slice-by-slice analyses using CNN training on publicly available cancer data. The dermoscopic images in the dataset are shown in Figure 12a–c.

The images are imported from the dataset using Keras’ preprocessed image data generation functions and resized to (224 × 224 × 3) with properly adjusted dimensions, width, and RGB channels. Other preprocessing steps involve rescaling, rotation degrees, zoom degrees, and mirroring. Subsequently, the data generator function is utilized to import the image dataset from the directory. Parameters such as probabilities are set, tested, validated, and defined, including the target size, batch size, and class mode for this function, and they are prepared using the specified architecture by adding a CNN layer. The photos are then divided into training and test datasets, consisting of 470 images for training and 203 images for testing. Table 6 shows the number of images in three specific categories.

**Figure 13:**
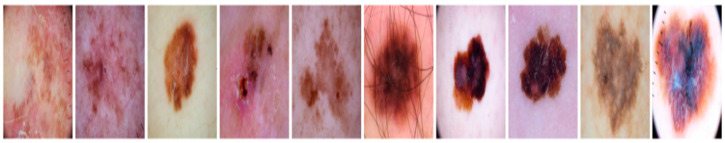
Training data for malignant class.

**Figure 14:**
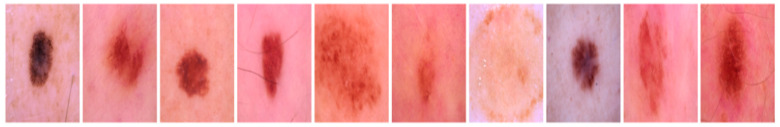
Training data for benign class.

**Figure 15:**
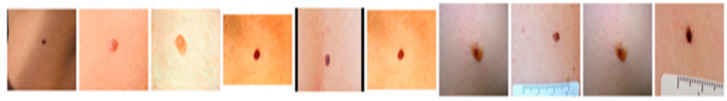
Training data for normal class.

The provided images belong to three different categories of training datasets, which include malignant, benign, and normal. Table 7 and Figure 13 depict the training data for the malignant class, comprising 178 images. Table 8 and Figure 14 display the training data for the benign class, consisting of 178 images. Table 9 and Figure 15 illustrate the training data for the normal class, comprising 114 images. Each image is characterized by its dimensions, width, and RGB channels, with a size of 224 × 224 × 3.

**Figure 16:**
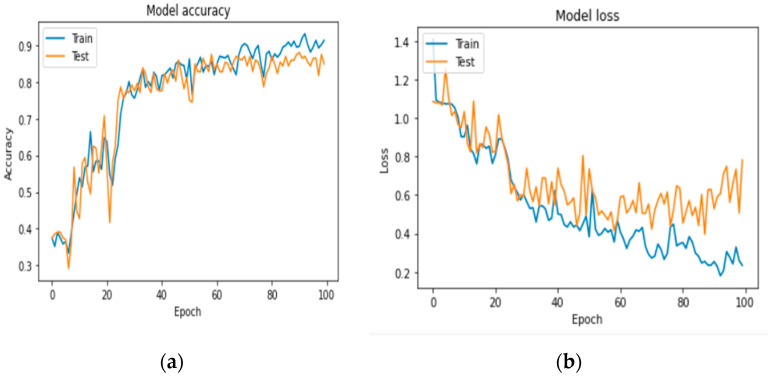
(**a**,**b**) AlexNet accuracy and loss curve as epochs increase.

The images were processed using the pre-learning AlexNet architecture consisting of eight-layer networks. Usually, pretraining classifies the image into 1000 objects. However, in our study, we divided them into three groups: benign, malignant, and elderly. Figure 16 shows a graph showing the exposure and loss of the pattern.

**Figure 17:**
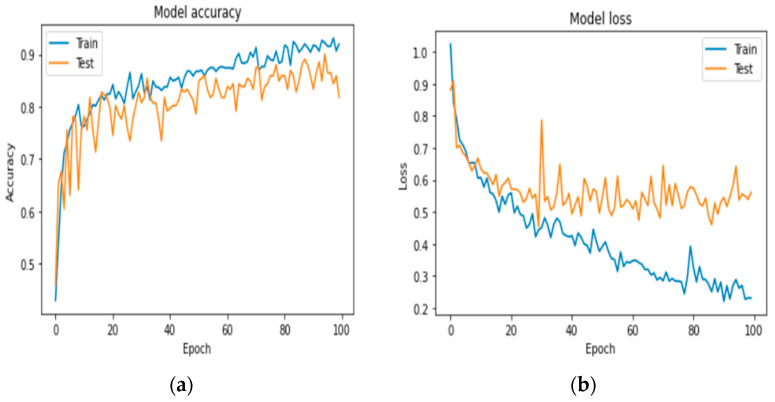
(**a**,**b**) LeNet accuracy and loss curve as epochs increase.

The image was made by learning from CNN architectures, particularly the LeNet-5 design, which is considered the best “input architecture” for neural networks. LeNet is simple and easy to use and also delivers great results. The previously trained network was able to separate the images into 10 different groups, but in this study, the images are divided into 3 groups: benign, malignant and normal. Figure 17 visually represents the exposure and loss of the pattern.

**Figure 18:**
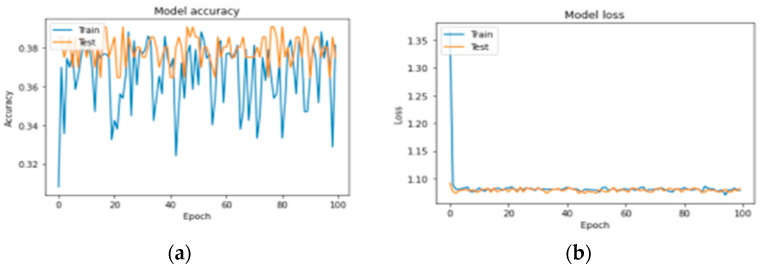
(**a**,**b**) VGG-16 accuracy and loss curve as epochs increase.

The images were fed into the pretraining CNN VGG-16 architecture, which consists of neural networks consisting of 16 layers. Most pretraining programs can separate images into 1000 different groups. However, in this study, the images were divided into three groups: benign, malignant, and normal. Figure 18 shows the accuracy and loss of the model.

As the amount of information decreases, the size of the convolution layer will be relatively small. As the number of layers in the convolution process increases, the complexity and time required also increases. This improvement allows CNN architectures to outperform pretrained models when processing certain data. Table 10 gives the layout of the proposed model.

**Figure 19:**
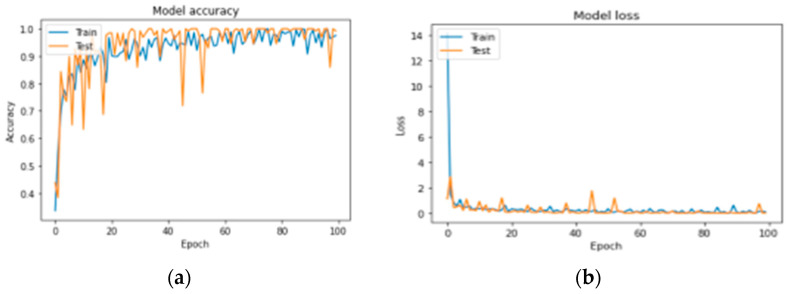
(**a**,**b**) Designed CNN network accuracy and loss curve as epochs increase.

Figure 19 presents a graph showing sample exposure and loss. The performance of each model is described over a training period of 100 epochs with a batch size of 32. Each period includes training accuracy and learning loss. Therefore, the proposed CNN architecture exhibits better accuracy and performance compared to the other previous learning models and will be used in future deployments. Table 11 provides information about the loss and accuracy of the proposed CNN architecture and the various CNN configurations.

**Figure 20:**
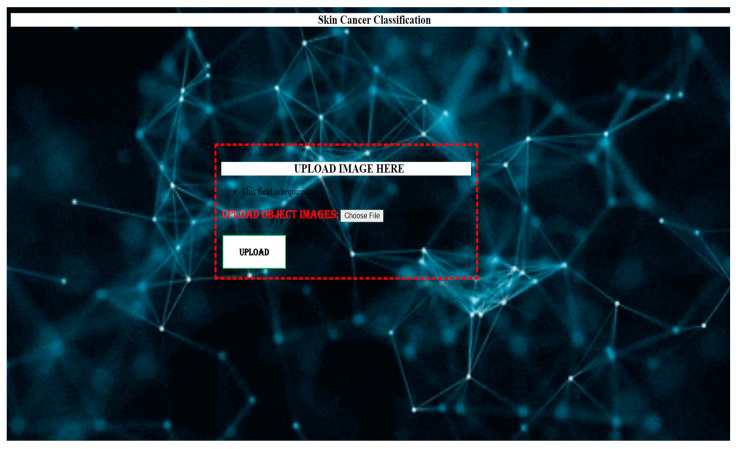
Homepage of the webpage.

**Figure 21:**
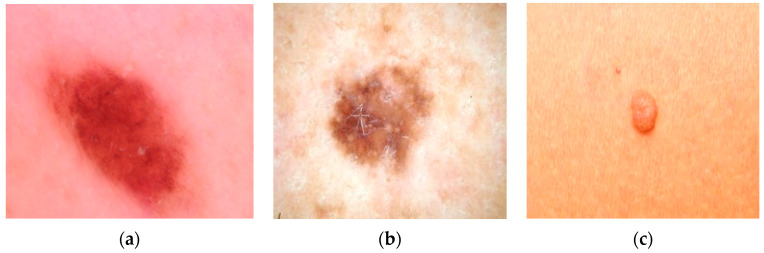
(**a**–**c**) Image uploading.

We first evaluated the accuracy and loss of performance evaluation models by deploying our models developed by VGG-16, AlexNet, LeNet, and keras.applications and by comparing these models with the same ISIC dataset. When all the models are trained with the same settings and using batch sizes of 32 and 100 epochs, the custom-built network achieves the highest accuracy of 91%, followed by AlexNet (84% accuracy) and LeNet (84% accuracy). VGG-16 achieved an accuracy of 37%. The models with higher accuracy also show lower losses. Private network lost 17%, AlexNet 78%, LeNet 59%, and VGG-16 108%. Figure 16, Figure 17 and Figure 18 show the learning curve of the training results. The ready-made model is included in the web application for more versatility. It is integrated into the Django framework to predict the results. The deep learning model is converted into a hierarchical file (“.h5 file”) and integrated into the Django framework to improve user experience and provide predictions on whether the uploaded image is malignant, benign, or normal. The front end is built using HTML and CSS, and the home page of the web application is shown in Figure 20. After opening the application, users will see this page. To send diagnostic images, the user clicks the “Select file” button and begins sending images, as shown in Figure 21.

**Figure 22:**
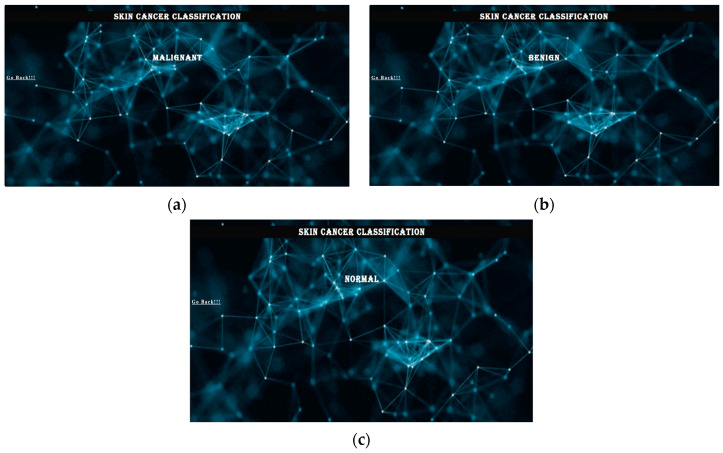
(**a**–**c**) Example result page for malignant, benign, and normal.

After successful loading, the program moves to another page and shows the results with the highest accuracy according to the desired model. To view the results, the user can view the results by clicking the “Get” button or by going back and clicking the “Back” button. Figure 22 shows the resulting page of the web application.

**Figure 23:**
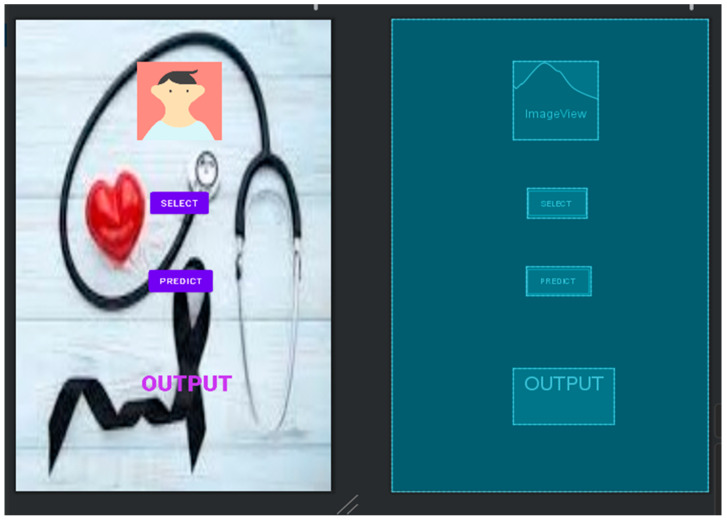
GUI for mobile application.

Click the Results button to see the results on your screen. If the user wants to go back, he must use the Back button. The online application is easy to use and accessible to everyone. TensorFlow Lite is a tool that allows developers to run models on mobile, embedded, and edge devices to enhance the capabilities of AI tools. TensorFlow Lite optimizes TensorFlow models into smaller, more compact, and more efficient AI models. Users can use predefined models from TensorFlow Lite for Android or create their own TensorFlow models and converting them to the TensorFlow Lite mode. Figure 23 shows the graphical user interface (GUI) of the mobile application coded in Java in Android Studio.

**Figure 24:**
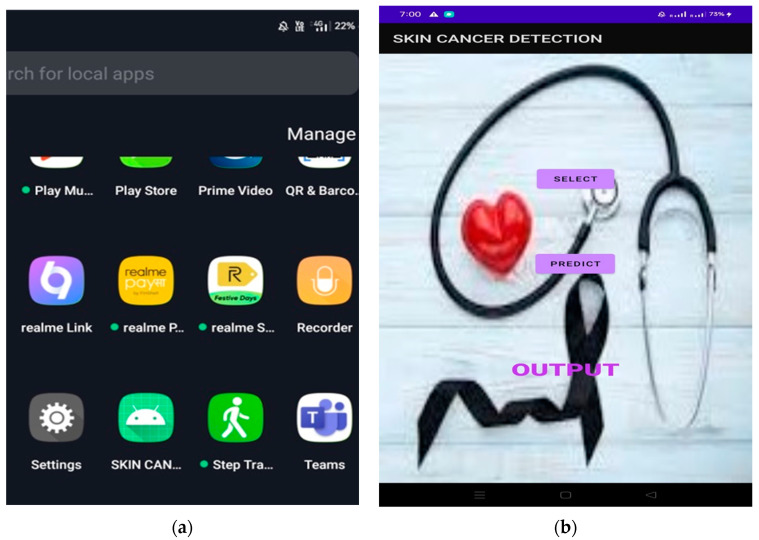
(**a**,**b**) Application installed in mobile and screen on mobile phone.

Use the TensorFlow Lite converter to transform the best models and place them in Android Studio. Shooting requires a Wi-Fi connection with your phone, and the results will appear on your mobile phone. Figure 24 shows a smartphone screen. The “Select” button allows you to select photos from your phone. A prediction function is used to predict outcomes based on a model that provides probabilities for each category. Select the prediction result with the best value and paste it into the result box.

**Figure 25:**
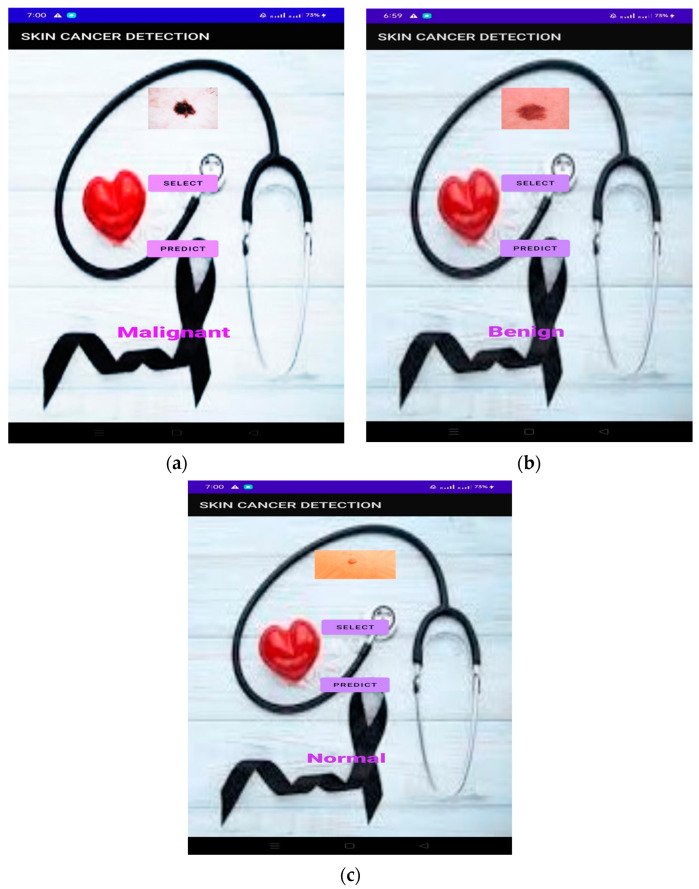
(**a**–**c**) Prediction of output in the mobile application for malignant, benign, and normal.

When an image is selected, the results are displayed in a text box. Android Studio then creates an .apk file and installs it on your phone using Build → Build → Build APK, as shown in Figure 24. Figure 25 shows the result of the prediction in the mobile application.

## 4. Conclusions

The proposed approach focused on classifying skin diseases as benign, malignant, or normal. In the obtained dataset, the convolutional neural network achieved 94% accuracy, while the SVM classifier achieved 86.6% accuracy. Instead of using traditional AI algorithms for skin disease detection, this approach introduced deep learning-based methods to automate feature extraction. When compared to existing AI systems, this proposed approach significantly improved accuracy and reduced errors. We compared the constructed CNN architecture with several CNN models, like Alexnet, LeNet, and VGG-16, to identify skin cancer symptoms. After training and testing these four well-known CNN models on the same ISIC dataset and evaluating their accuracy and loss, the developed CNN architecture achieved an accuracy of 94% with a loss of 17% when provided with identical data inputs. The CNN model, which consistently showed the highest accuracy across various tests, was chosen as the dermatology test. Therefore, the proposed CNN model can be incorporated into web and mobile applications to help diagnose skin diseases. This method provides results that are faster, more accurate, and easier for non-dermatologists to use than the traditional methods. Additional plans include continuing to work on the app to improve the user experience, fixing bugs such as batch size and time to test the model’s response to these changes, and exploring opportunities to feed models with larger datasets in future work.

## Figures and Tables

**Table 1:** Segmentation parameters.

S. No.	Images	Threshold	Entropy
1	1	164	4.415
2	2	160	3.977

**Table 2:** Mean and standard deviation of elements extricated from threatening and harmless pictures.

S. No.	Features	Benign	Malignant
1	Mean	67.9958 ± 18.841	75.926 ± 25.194
2	Median	66.85 ± 22.43216	69.69 ± 28.15571
3	Area	6727.06 ± 3149.772	13,009.76 ± 6649.319
4	Perimeter	289.52 ± 92.624	405.02 ± 119.0192
5	Coefficient of variation	0.0815 ± 0.009398	0.00204 ± 0.016354
6	Inverse difference moment	0.205417 ± 0.070323	0.1841 ± 0.0543
7	Sum average	139.131 ± 47.795	150.46 ± 60.2164
8	Gray level variance	20.23 ± 15.1	8.221 ± 5.54
9	Zone size entropy	−7.2965 ± 0.21483	−7.51093 ± 0.29246
10	Difference entropy	−3.7532 ± 0.76304	−3.9180 ± 0.71125
11	Histogram width	69.85 ± 17.658	72.852 ± 22.28
12	Maximum gray level intensity	162.3 ± 18.04381	185.46 ± 31.8422
13	Minimum gray level intensity	31.5 ± 15.13106	21.6078 ± 18.0431
14	Coefficient of variation	0.0815 ± 0.009398	0.00204 ± 0.016354
15	Inverse difference moment	0.205417 ± 0.070323	0.1841 ± 0.0543
16	Sum average	139.131 ± 47.795	150.46 ± 60.2164
17	Gray level variance	20.23 ± 15.1	8.221 ± 5.54
18	Zone size entropy	−7.2965 ± 0.21483	−7.51093 ± 0.29246
19	Difference entropy	−3.7532 ± 0.76304	−3.9180 ± 0.71125
20	Histogram width	69.85 ± 17.658	72.852 ± 22.28
21	Maximum gray level intensity	162.3 ± 18.04381	185.46 ± 31.8422

**Table 3:** SVM classifier output.

Classification Report of Support Vector Classifier					
S. No.		Precision	Recall	F1-Score	Support
1.	Benign	0.82	0.93	0.87	15
2.	Malignant	0.92	0.80	0.86	15

**Table 4:** The classifier’s classification report.

S. No.		Precision (%)	Recall (%)	F1 Score (%)
1.	Benign	82	93	87
2.	Malignant	92	80	86

**Table 5:** Model evaluation.

S. No.	Parameters	Overall %
1	Accuracy	86.6
2	Sensitivity	82.3
3	Specificity	92.3

**Table 6:** Image count of three different classes.

S. No.		Image Count	Classes
1.	Training	470	3
2.	Testing	203	3

**Table 7:** Training data for malignant class.

S. No.	Training Data for Malignant	
	Images in: Data/Train/Malignant	
1.	images_count:	178
2.	min_width:	224
3.	max_width:	224
4.	min_height:	224
5.	max_height:	224

**Table 8:** Training data for benign class.

S. No.	Training Data for Benign	
	Images in: Data/Train/Benign	
1.	images_count:	178
2.	min_width:	224
3.	max_width:	224
4.	min_height:	224
5.	max_height:	224

**Table 9:** Training data for normal class.

S. No.	Training Data for Normal	
	Images in: Data/Train/Normal	
1.	images_count:	114
2.	min_width:	224
3.	max_width:	224
4.	min_height:	224
5.	max_height:	224

**Table 10:** The architecture of the proposed CNN model.

	Model: “Sequential”		
S. No.	Layer (Type)	Output Shape	Parameter
1.	conv2d (Conv2D)	(None, 56, 56, 32)	896
2.	activation (Activation)	(None, 56, 56, 32)	0
3.	max_pooling2d (MaxPooling2D)	(None, 28, 28, 32)	0
4.	conv2d_1 (Conv2D)	(None, 12, 12, 64)	51,264
5.	activation (Activation)	(None, 12, 12, 64)	0
6.	max_pooling2d_1 (MaxPooling2D)	(None, 6, 6, 64)	0
7.	flatten (Flatten)	(None, 2304)	0
8.	dense (Dense)	(None, 38)	87,590
9.	dropout (Dropout)	(None, 38)	0
10.	Dense_1 (Dense)	(None, 3)	117

**Table 11:** Misfortune and exactness for the arranged CNN organization and different CNN designs.

Performance of the Trained Results								
S. No.	Models	Database	Epochs	Batch Size	Training Accuracy (%)	Training Loss (%)	Testing Accuracy (%)	Testing Loss (%)
1.	ALEX NET	ISIC	100	32	91	23	84	78
2.	LENET	ISIC	100	32	90	22	81	59
3.	VGG16	ISIC	100	32	38	107	37	108
4.	DESIGNED NETWORK	ISIC	100	32	94	15	91	17

## Data Availability

Data sharing not applicable.
